# Serum resistin positively correlates with serum lipids, but not with insulin resistance, in first-degree relatives of type-2 diabetes patients: an observational study in China

**DOI:** 10.1097/MD.0000000000006622

**Published:** 2017-04-21

**Authors:** Xiao-hong Niu, Li Li, Jun-yan Li, Qi Song, Miao-miao Jin, Jin-xia Liu

**Affiliations:** a Department of Endocrinology; b Clinical Laboratory, Heji Hospital Affiliated to Changzhi Medical College, Changzhi, China.

**Keywords:** first-degree relatives, hyperlipidemia, insulin resistance, resistin, type-2 diabetes mellitus

## Abstract

To investigate whether serum resistin correlated with hypertension, obesity, dyslipidemia, or insulin resistance (IR) in Chinese type 2 diabetes mellitus (T2DM) patients and their first-degree relatives (DFDRs) in a case–control observational study.

We determined the serum levels of resistin, plasma lipids, glucose, and insulin, and performed clinical assessments of hypertension, obesity, and IR for 42 T2DM patients, 74 of their DFDRs, and 51 healthy control participants with no family history of T2DM (NC group). The biochemical and clinical variables were compared between the 3 groups, and relationships between serum resistin and the other variables were evaluated using a Pearson correlation analysis.

Significant trends were observed in the triglyceride, HbA1c, and resistin levels, in which the values observed in the DFDR group were intermediate to those of the T2DM and NC groups (*P* < .05 for all). A stratified analysis revealed significant trends in the resistin level and scores for homeostasis model assessment (HOMA) indexes for IR and insulin sensitivity in women and in the HbA1c and resistin levels in men (*P* < .05 for all), with DFDR subjects exhibiting intermediate values. The Pearson analysis showed that serum resistin positively correlated with total cholesterol and low-density lipoprotein cholesterol in the DFDR group only (*P* < .05 for both), and that resistin did not correlate significantly with HOMA indexes, blood glucose, insulin, HbA1c, triglyceride, high-density lipoprotein cholesterol, BMI, waist or hip circumference, or blood pressure.

Our results suggest that elevated serum resistin might contribute to an increased risk of hyperlipidemia in DFDRs of Chinese T2DM patients.

## 1 Introduction

Type-2 diabetes mellitus (T2DM) is a major public health problem in industrialized nations and many developing countries. In 2012, approximately 3.7 million deaths were caused by T2DM and hyperglycemia-related complications worldwide.^[1]^ The incidence of T2DM is increasing on a global scale, placing an equally increasing burden on the health care systems of countries most affected by this public health crisis.^[1,2]^ Although studies have shown that T2DM is associated with insulin resistance (IR), obesity, cardiovascular disease (CVD), hypertension, dyslipidemia, and nonalcoholic fatty liver disease,^[3–7]^ the biochemical links through which these conditions are induced by the dysregulation of homeostatic processes remain unclear.

Resistin is an adipokine that has been linked to the development of T2DM in rodent models. The overexpression of resistin from adipocytes induced the development of IR and dyslipidemia in healthy mice.^[8]^ Other studies have shown that the loss of resistin expression improved blood glucose maintenance and insulin sensitivity (IS) in diet-induced obese mice and leptin knockout mice, which develop both obesity and IR.^[9,10]^ Although human resistin shares only 60% amino acid sequence identity with that of mouse resistin^[11]^ and is expressed primarily by macrophages,^[12,13]^ rather than adipocytes, patients with T2DM often exhibit elevated resistin levels.^[14]^ In addition, clinical investigations have reported a positive correlation between the serum level of resistin and the severity of IR assessed according to the homeostatic model assessment (HOMA).^[15–17]^ Elevated resistin is also a risk factor for CVD and all-cause mortality in T2DM patients.^[18]^ However, other studies have been unable to confirm an association between serum resistin and T2DM, and there is a lack of clear evidence of a biological mechanism through which resistin contributes to the development of IR in humans.

First-degree relatives of T2DM patients (first-degree relatives of diabetic patients [DFDRs]) have an increased risk of developing hyperglycemia,^[19]^ and recent genomic studies have identified T2DM susceptibility loci in humans.^[20,21]^ Given the similarity of the genetic background of T2DM patients with that of their DFDRs, we reasoned that the serum level of resistin in the DFDRs of T2DM patients might also correlate with IS and perhaps various other features of the metabolic syndrome. To determine whether such correlations do indeed exist, we compared the serum level of resistin and various markers of IR, dyslipidemia, and obesity between T2DM patients, their DFDRs, and healthy control subjects. Our findings suggest that, in DFDRs of T2DM patients, sex has a significant influence on markers of IR, and that the level of serum resistin correlates with certain markers of hyperlipidemia.

## 2 Material and methods

### 2.1 Study population

We performed an observational study from January 2014 to June 2015, in which T2DM patients treated in our department, their siblings and/or children, and healthy volunteers with no family history of T2DM were enrolled in the T2DM, DFDR, and normal control (NC) groups, respectively. The exclusion criteria for DFDR and NC participants were an abnormal result for the oral glucose tolerance test (OGTT); a history of abnormal OGTT results; currently using drugs affecting glucose and/or lipid metabolism; abnormal hepatic and/or renal laboratory findings; chronic or acute infection; and any type of malignancy and currently diagnosed with an autoimmune or other systemic disease.

The inclusion criteria for T2D patients were as follows: diagnosis of diabetes type 2 (World Health Organization criteria of 1999); onset age 18 to 65 years; disease onset within 6 months; and not yet being treated (including diet, exercise, and drugs). Exclusion criteria were type 1 diabetes, secondary diabetes, diabetes in pregnancy, and gestational diabetes mellitus; liver and kidney dysfunction; and patients with mental illness and cancer.

Participants in the DFDR and NC groups were matched based on sex and age. Our study was approved by the Institutional Review Board of Heji Hospital Affiliated to Changzhi Medical College (approval no. 2007IRB(S)02), and all of the participants provided written informed consent prior to enrollment. Our study was conducted in accordance with the Declaration of Helsinki regarding ethical standards for research involving human subjects.

### 2.2 Laboratory and clinical assessments

All participants underwent an OGTT after an overnight 8- to 10-hour fasting period. Participants received 75 g of glucose orally, and blood samples were collected at 0, 30, and 120 minutes following glucose treatment (PGT). Blood glucose concentrations were measured using the glucose oxidase method. The HOMA indexes were calculated based on the fasting blood levels of glucose (FBG) and insulin (FBI) at 0 minute PGT. The HOMA-IR was calculated as HOMA-IR = BI (mIU/L) × (FBG (mmol/L)/22.5), as described previously.^[22]^ The HOMA-IS was calculated as HOMA-IS = 1/[FBG (mmol/L) × FBI (mIU/L)], as described previously.^[23]^ To assess pancreatic β cell function, the HOMA-β was calculated as HOMA-β = 20 × [FBI (mIU/L)/(FBG (mmol/L)–3.5)], as described previously.^[24]^ Blood samples collected at 0 minute PGT were subjected to additional analyses. Triglycerides and total cholesterol (TC) were measured using routine enzymatic methods. High-density lipoprotein cholesterol (HDL-C) was determined after apoB-lipoprotein precipitation, and low-density lipoprotein cholesterol (LDL-C) was calculated using the Friedewald formula.^[25]^ Insulin was measured using an enzyme-linked immunosorbent assay (ELISA; Boster, Wuhan, China). Glycated hemoglobin, HbA1c, was measured in a Roche COBAS-400 automated biochemical analyzer (Roche Applied Science, Penzberg, Germany) using EDTA-K2 (Sigma–Aldrich, St. Louis, MO) as an anticoagulant. Resistin was measured using an ELISA (Boster, Wuhan, China). Systolic and diastolic blood pressure (BP) were measured at each time point using an electronic BP monitor. Body-mass index (BMI) was calculated as mass in kilograms divided by surface area in square meters. Waist and hip circumferences were also recorded. Both the study personnel and the participants were blinded to group assignment when these data were recorded, and all of the participants in each group completed the abovementioned analyses.

### 2.3 Statistical analysis

The statistical analysis was performed using the SPSS, version 13.0, software (IBM, Armonk, NY). To evaluate sample size, statistical power was estimated using a 2-sided α = 5%, and the calculations indicated that, at a level of statistical power = 80%, a combined total of ≥76 participants were required to detect HOMA-IR at a mean and standard deviation of 3.0 ± 2.6 in the T2DM group (n ≥ 38) and 1.6 ± 1.4 in the control group (n ≥ 38). Categorical variables are reported as number and percent, and continuous variables are reported as the mean ± standard deviation. Categorical variables were compared using a chi-squared analysis. Normally distributed continuous variables were subjected to an analysis of variance, and those lacking a normal distribution were compared using the Wilcoxon rank sum test. Pairwise comparisons were performed using *t* tests to evaluate intergroup differences. A stratified analysis was performed to investigate the effects of sex on age, biochemical variables, and clinical assessments. The statistical relationships between the serum level of resistin and the study variables were evaluated using a Pearson correlation analysis. The level of statistical significance was set at *P* < .05.

## 3 Result

### 3.1 Demographic characteristics

The demographic characteristics of the cohort are presented in Table 1. Our final analysis included 42, 74, and 51 participants in the T2DM, DFDR, and NC groups, respectively. The mean age of the participants was 42.7 ± 12.3 years. Participants in the T2DM group were significantly older (49.5 ± 13.6 years) than participants in the DFDR and NC groups (41.4 ± 10.5 and 39.0 ± 11.7 years, respectively; *P* < .05). The difference in age between the T2DM group and the DFDR and NC groups is consistent with the chronic disease course of T2DM and our study design, which included the selection of both the siblings and offspring of T2DM patients and matching of the DFDR and NC groups based on age and sex. Of the 167 total participants, 98 (58.7%) were women, and no significant difference in the proportion of women was observed between the 3 study groups (*P* > .05). The stratified analysis showed that women in the T2DM group were significantly older than those in the DFDR and NC groups (*P* < .05 for both; Table 2), whereas the ages of the women in the DFDR and NC groups did not differ significantly. By contrast, no significant differences were observed in the men in the T2DM, DFDR, and NC groups (*P* > .05).

**Table 1:**
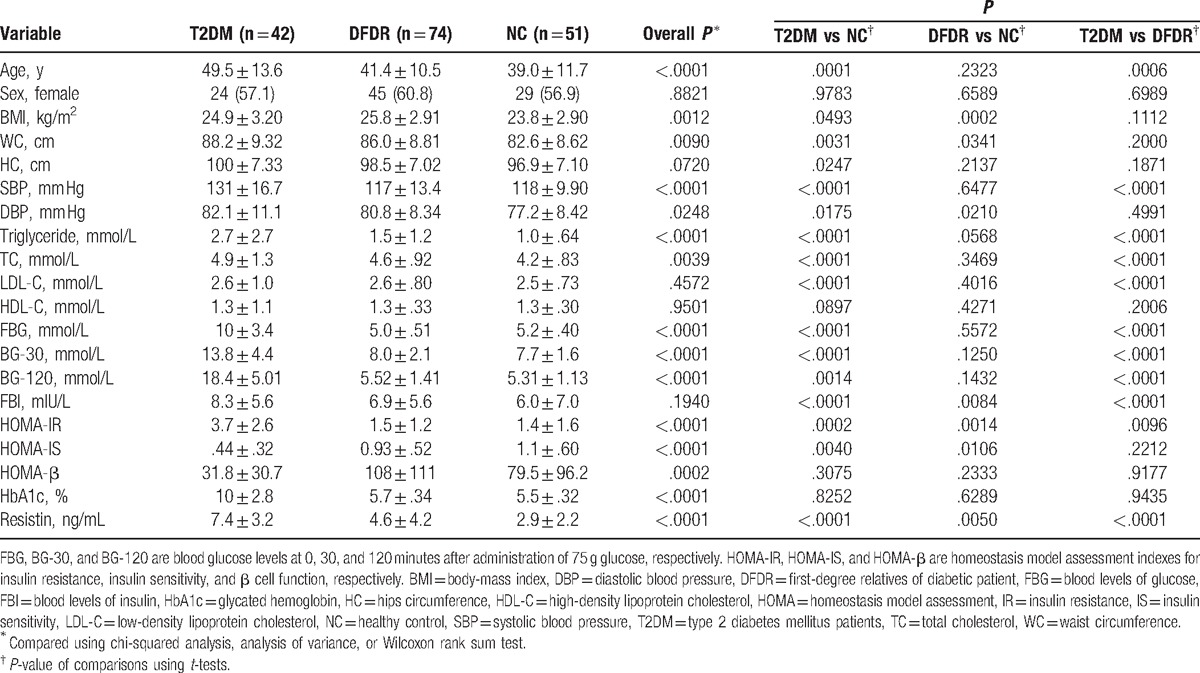
Demographic, clinical, and biochemical characteristics of the study participants.

**Table 2:**
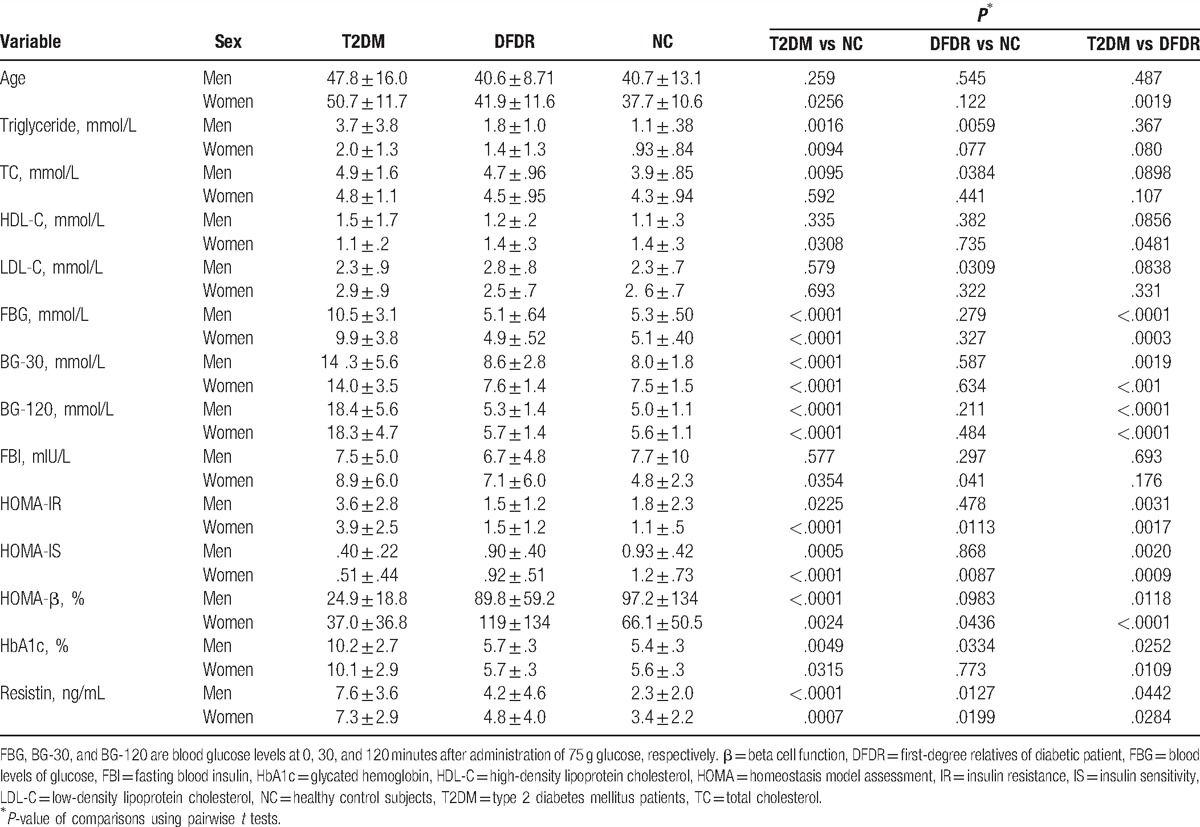
Stratified comparisons of the demographic, clinical, and biochemical characteristics based on sex.

### 3.2 Markers of obesity

The mean BMI was highest in the DFDR group (25.8 ± 2.91 kg/m^2^, *P* < .05) and lowest in the NC group (23.8 ± 2.90 kg/m^2^; Table 1). The BMI of the NC group was significantly lower than that of the T2DM and DFDR groups (*P* < .05 for both), but the BMI of the T2DM and DFDR groups did not differ significantly (*P* > .05). Waist circumference was significantly larger in the T2DM and DFDR groups, compared with that in the NC group (*P* < .05 for both), whereas the waist circumference of the T2DM and DFDR groups did not differ significantly (*P* > .05). Hip circumference in the T2DM group was significantly larger than that in the DFDR and NC groups (*P* < .05 for both), whereas hip circumference in the DFDR and NC groups did not differ significantly (Table 1). These data suggest that the risk of obesity in DFDRs of T2DM patients might be greater than that in people of similar age with no family history of T2DM. In the stratified analysis, no clear pattern was observed in the differences between the BMI, waist circumference, and hip circumference of men or those of women (data not shown).

### 3.3 Blood pressure

Overall, no clear trend was observed in BP across the 3 study groups. Both systolic and diastolic BP were highest in the T2DM group (131 ± 16.7 and 82.1 ± 11.1 mm Hg, respectively; *P* < .05 for both), compared with those of the DFDR and NC groups (Table 1). However, systolic BP did not differ significantly between the DFDR and NC groups, and the diastolic BP in the T2DM group did not differ significantly, compared with that of the T2DM group (*P* > .05 for both; Table 1). In the stratified analysis, no clear pattern was observed in the differences between the systolic or diastolic BP of men or those of women (data not shown). These data suggest that risk of hypertension in DFDRs of T2DM patients is similar to that in people of similar age with no family history of T2DM.

### 3.4 Markers of dyslipidemia

We also examined the serum lipid profile of participants (Table 1). A significant trend was observed in the serum triglyceride level, which was highest in the T2DM group (2.7 ± 2.7 mmol/L), compared to that of the DFDR and NC groups (1.5 ± 1.2 and 1.0 ± .64 mmol/L, respectively, *P* < .05 for both). Pairwise comparisons of the triglyceride levels revealed significant differences between all of the study groups (*P* < .05 for all). The TC level was also highest in the T2DM group (4.9 ± 1.3 mmol/L, *P* < .05), compared to the DFDR and NC groups (Table 1). Although the TC level in the NC group was significantly lower than that of the T2DM and DFDR groups (*P* < .05 for both), the TC level in the T2DM and DFDR groups were similar (*P* > .05). Significant differences in LDL-C and HDL-C were not observed (*P* > .05 for all; Table 1). These data suggest that male DFDRs of T2DM patients might have a greater risk of dyslipidemia than people with no family history of T2DM.

In the stratified analysis, significant differences in the levels of triglycerides and TC were observed between men in the T2DM and NC groups and between the men in the DFDR and NC groups, whereas the levels of triglycerides and TC were similar between the men in the T2DM and DFDR groups (Table 2). Among the women in our cohort, only those in T2DM and NC groups had triglyceride levels that differed significantly (*P* < .05), and none of the women in any of the 3 study groups exhibited significant differences in TC (Table 2). Although no clear pattern was observed in HDL-C and LDL-C based on sex. The HDL-C level in women in the T2DM group was significantly lower than that of women in the DFDR and NC groups (*P* < .05 for both), but HDL-C in men did not differ significantly (*P* > .05 for all). The LDL-C level in men in the DFDR group was significantly greater than that in men in the T2DM and NC groups (*P* < .05 for both), whereas the HDL-C in men in the T2DM group did not differ significantly from that of men in the NC group (*P* > .05). No significant differences were observed in the LDL-C levels in women (*P* > .05 for all). Despite the lack of a clear pattern in lipid profiles, these data suggest that the risk of dyslipidemia in male DFDRs of T2DM patients is greater than that of female DFDRs.

### 3.5 Glycemic parameters

As expected, the mean FBG was highest in the T2DM group (10 ± 3.4 mmol/L, *P* < .05), compared to the DFDR and NC groups (5.0 ± .51 and 5.2 ± .40 mmol/L, respectively; Table 1). However, the FBG of the DFDR group did not differ significantly from that of the NC group alone (*P* > .05). This pattern in blood glucose levels was also observed at the 30 and 120 minutes PGT time points (Table 1). The FBI levels did not differ significantly between the 3 study groups (*P* > .05). The HOMA index values reflected the results of the OGTT for the study groups. As expected, the HOMA-IR was highest for the T2DM group (3.7 ± 2.6, *P* < .05), compared to that of the DFDR and NC groups (1.5 ± 1.2 and 1.4 ± 1.6, respectively; Table 1), and the HOMA-IS of the T2DM group (.44 ± .32, *P* < .05) was significantly lower than that of the DFDR and NC groups (.93 ± .52 and 1.1 ± .60), which were statistically similar (*P* > .05; Table 1). The HOMA-β was also lowest in the T2DM group (*P* < .05), and the HOMA-β of the DFDR and NC groups did not differ significantly (*P* > .05; Table 1). Levels of HbA1c were consistent with the results of the OGTT. The HbA1c level in the T2DM group was significantly higher than that in the DFDR and NC groups (*P* < .05 for both), which did not differ significantly (*P* > .05; Table 1).

In the stratified analysis, differences in the OGTT blood glucose levels for men and women were consistent with the findings of the overall analysis in Table 1, with higher blood glucose values in the T2DM group, compared to the DFDR and NC groups, and no significant difference between those of the DFDR and NC groups (Table 2). Differences in the HOMA indexes for men were also consistent with the findings of the overall analysis. However, a clear trend was observed in the results for the HOMA indexes for women, with significant differences in HOMA indexes observed between the 3 of the study groups (*P* < .05 for all; Table 2). In women, the level of HbA1c was consistent with the blood glucose level, with the HbA1c level in female T2DM patients being significantly higher (*P* < .05) than that of women in the DFDR and NC groups, whereas the HbA1c level in the DFDR and NC groups did not differ significantly (*P* > .05; Table 2). In men, however, significant differences in the HbA1c level were observed between the three study groups (*P* < .05 for all), with the highest and lowest levels of HbA1c observed in the T2DM and NC groups, respectively. The FBI level was significantly higher in women in the T2DM and DFDR group (*P* < .05 for both), compared with that of women in the NC group, and the FBI level in the T2DM and DFDR groups did not differ significantly (*P* > .05).

These data collectively suggested that the DFDRs of T2DM patients might be at greater risk of IR than people with no family history of T2DM. However, the data also suggest that the predictive value of HOMA indexes might be greater for female DFDRs than for male DFDRs of T2DM patients, given the clear trend in the values of those parameters in women. By contrast, the predictive value of the HbA1c level might be greater for male DFDRs than for female DFDRs, given the clear trend observed in HbA1c in men.

### 3.6 Serum resistin levels

A significant trend was observed in the serum level of resistin. The resistin level was highest in the T2DM group (7.4 ± 3.2 ng/mL, *P* < .05) and lowest in the NC group (2.9 ± 2.2 ng/mL), whereas the level of resistin in the DFDR group 4.6 ± 4.2 ng/mL differed significantly from that in the T2DM and NC groups (Table 1). In the stratified analysis, this trend was observed in both men and women (Table 2), with significant differences in resistin level between the 3 study groups. Previous studies have reported higher resistin levels in patients with T2DM, compared to non-T2DM subjects.^[14,26]^ These data suggest that DFDRs of T2DM patients might also have higher serum resistin levels than people with no family history of T2DM, and that the level of serum resistin may increase incrementally in the progression of T2DM.

### 3.7 Correlational analysis

The results of the Pearson correlation analysis of the statistical relationship between the serum level of resistin and the demographic, clinical, and biochemical variables are presented in Table 3. The results showed that the serum resistin level did not significantly correlate with age, BP, markers of obesity, insulin level, blood glucose or HbA1c level, or the HOMA indexes in any of the 3 study groups. However, serum resistin did significantly correlate with TC (*R* = .343, *P* = .0028) and LDL-C (*R* = .310, *P* = .0072) in the DFDR group, but not in the T2DM or NC groups (*P* > .05 for both). The serum resistin level did not correlate with level of triglycerides or HDL-C (*P* > .05 for all) in any of the study groups. These data suggest that an increased serum resistin level might be a risk factor for increased levels of LDL-C and TC, which are major determinants of hyperlipidemia.

**Table 3:**
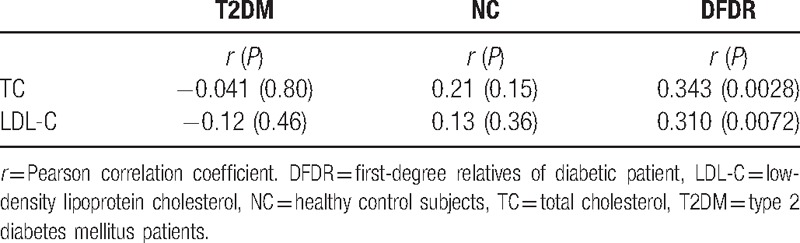
Pearson correlation analysis of serum lipid and resistin levels in the study groups.

## 4 Discussion

We performed a cross-sectional analysis of age, sex, serum resistin level, BP, various markers of obesity and dyslipidemia, serum insulin, fasting blood glucose, OGTT results, and HOMA indexes in T2DM patients, their DFDRs, and healthy control subjects with no family history of T2DM to investigate whether DFDRs of T2DM patients exhibit variation in these variables, compared to T2DM patients and healthy people who are not genetically predisposed to T2DM. Significant trends were observed in the triglyceride, HbA1c, and resistin levels in which the values observed in the DFDR group were intermediate to those of the T2DM and NC groups (*P* < .05 for all; Table 1). The stratified analysis revealed significant trends in HOMA-IR, HOMA-IS, and resistin in women and in HbA1c and resistin in men, with DFDR subjects exhibiting values intermediate to those of T2DM and NC subjects (Table 2). However, the Pearson analysis showed that serum resistin positively correlated with TC and LDL-C in the DFDR group only, and that resistin did not correlate significantly with the HOMA indexes, OGTT results, FBI, HbA1c, triglyceride, HDL-C, waist or hip circumference, or BP in any group in our cohort. Our results suggested that elevated serum resistin might contribute to an increased risk of hyperlipidemia in DFDRs of Chinese T2DM patients.

Elevated levels of resistin have been reported in T2DM patients,^[27–29]^ and previous studies have reported associations between serum resistin and HOMA-IR.^[17,28]^ We found that resistin levels in T2DM patients and their DFDRs were significantly higher than that in participants with no family history of T2DM, with the highest levels of resistin observed in the T2DM group (Table 1), and this trend in resistin levels was not significantly affected by sex (Table 2). However, we also found no significant correlation between resistin and HOMA-IR in either T2DM or DFDR groups, which is consistent with the findings of Youn et al,^[29]^ who published the seminal report of elevated resistin in T2DM patients. Our DFDR group had larger waist and hip circumference than the NC group, and they had higher BMI than both the T2DM and NC groups. Therefore, our data regarding elevated levels resistin in the DFDR group are partially consistent with the findings of a Silha et al,^[28]^ who reported elevated resistin in nondiabetic obese subjects. However, Silha et al also found that resistin correlated with HOMA-IR. Such inconsistencies in the findings of the role of resistin in IR and T2DM have plagued researchers since the discovery of elevated resistin levels in T2DM patients. However, to the best of our knowledge, this is the first report of a correlation between increased serum resistin and markers of hyperlipidemia in the DFDRs of T2DM patients.

A previous cell culture study showed that resistin treatment increased the intracellular level of triglycerides and cholesterol species by 25% and 18%, respectively, and stimulated the production of lipoprotein particles in human hepatocytes.^[30]^ In our overall descriptive analysis, we observed higher levels of triglycerides and TC in the T2DM and DFDR groups (*P* < .05). Although no clear trend in serum lipid profile was revealed in the stratified analysis, men in the T2DM and DFDR groups had higher triglyceride and TC levels (*P* < .05), and men in the DFDR group had higher LDL-C (*P* < .05), whereas women in the T2DM and DFDR groups had lower HDL-C. These data suggest that sex might exert significant influences on serum lipids in T2DM patients and their DFDRs. Studies in Japan found that a single-nucleotide polymorphism (−420) in the promoter region of the human resistin gene^[31]^ correlated with increased serum resistin and reduced HDL-C in nondiabetic subjects,^[32]^ and serum resistin was higher in women with the −420 mutation. We did not screen for the −420 mutation in our cohort, but our stratified analysis indicated that the serum resistin level was unaffected by sex. Hyperlipidemia is a risk factor CVD. Therefore, our results suggest that resistin might contribute to an increased risk of CVD in DFDRs of T2DM patients.

Although we did not find a correlation between resistin and blood glucose levels, HbA1c, or the HOMA indexes, noteworthy trends were revealed in the stratified analysis based on sex (Table 2). Although neither men nor women in the DFDR group exhibited blood glucose levels in the OGTT that were significantly higher than those of the NC group, significant trends in the HOMA indexes existed among the women in our study, with DFDR women exhibiting scores intermediate to that of the T2DM and NC groups (*P* < .05 for all). A significant trend in HbA1c level existed among our male subjects, with DFDR men exhibiting an HbA1c level intermediate to that of the T2DM and NC men (*P* < .05 for all). Significantly higher FBI in DFDR women, compared to NC women, likely contributed to the observed trend in HOMA indexes. Our data did not indicate a possible explanation for the higher HbA1c levels observed in DFDR men, compared to NC men. A recent study reported elevated resistin and HbA1c levels in obese nondiabetic subjects with IR. However, the HbA1c level in DFDRs of T2DM patients in our study was <7%, and was lower than that reported by El-Shal et al^[33]^ for the obese nondiabetic subjects with IR, whereas resistin levels in our DFDR subjects were higher than those reported by El-Shal et al.

Our findings are subject to certain limitations. Although we performed an analysis of statistical power (80%), a much larger study sample would still likely provide more reliable results than those obtained from our relatively small sample (N = 167). Therefore, future studies with larger study populations are required to further investigate the effects of biochemical risk factors for T2DM in DFDRs of T2DM patients. All of our participants were Han Chinese who resided in the Changzhi area, which might limit the generalization of our findings to other populations. We did not perform a correlation analysis of the stratified data because we believed the small sizes of the subgroups did not warrant this. Therefore, we are unable to rule out sex as a possible confounder in the Pearson analysis. Our results highlight the need for more rigorous study designs that consider sex, obesity, and HOMA index status with regard to group assignment to minimize or isolate potential confounders associated with sex and the various components of metabolic syndrome.

## References

[1] WHO. Global Report on Diabetes. 2016;Geneva, Switzerland: World Health Organization Press, 1–88.

[2] Caspersen CJ Thomas GD Boseman LA Aging, diabetes, and the public health system in the United States. Am J Public Health 2012;102:1482. 22698044 10.2105/AJPH.2011.300616 PMC3464829

[3] Alberti K Eckel R Grundy S Harmonizing the metabolic syndrome: a joint interim statement of the International Diabetes Federation Task Force on Epidemiology and Prevention; National Heart, Lung, and Blood Institute; American Heart Association; World Heart Federation; International Atherosclerosis Society; and International Association for the Study of Obesity. Circulation 2009;120:1640. 19805654 10.1161/CIRCULATIONAHA.109.192644

[4] Feng R-N Zhao C Wang C BMI is strongly associated with hypertension, and waist circumference is strongly associated with type 2 diabetes and dyslipidemia, in Northern Chinese Adults. J Epidemiol 2012;22:317. 22672914 10.2188/jea.JE20110120 PMC3798650

[5] Portillo-Sanchez P Bril F Maximos M High prevalence of nonalcoholic fatty liver disease in patients with type 2 diabetes mellitus and normal plasma aminotransferase levels. J Clin Endocrinol Metab 2015;100:2231. 25885947 10.1210/jc.2015-1966 PMC6287506

[6] Perry RJ Samuel VT Petersen KF The role of hepatic lipids in hepatic insulin resistance and type 2 diabetes. Nature 2014;510:84. 24899308 10.1038/nature13478 PMC4489847

[7] Chehade J Gladysz M Mooradian A Dyslipidemia in type 2 diabetes: prevalence, pathophysiology, and management. Drugs 2013;73:327. 23479408 10.1007/s40265-013-0023-5

[8] Sato N Kobayashi K Inoguchi T Adenovirus-mediated high expression of resistin causes dyslipidemia in mice. Endocrinology 2005;146:273–9. 15471967 10.1210/en.2004-0985

[9] Banerjee RR Rangwala SM Shapiro JS Regulation of fasted blood glucose by resistin. Science 2004;303:1195–8. 14976316 10.1126/science.1092341

[10] Qi Y Nie Z Lee YS Loss of resistin improves glucose homeostasis in leptin deficiency. Diabetes 2006;55:3083–90. 17065346 10.2337/db05-0615

[11] Ghosh S Singh A Aruna B The genomic organization of mouse resistin reveals major differences from the human resistin: functional implications. Gene 2003;305:27. 12594039 10.1016/s0378-1119(02)01213-1

[12] Patel L Buckels A Kinghorn I Resistin is expressed in human macrophages and directly regulated by PPAR gamma activators. Biochem Biophys Res Commun 2003;300:472. 12504108 10.1016/s0006-291x(02)02841-3

[13] Fain J Cheema P Bahouth S Resistin release by human adipose tissue explants in primary culture. Biochem Biophys Res Commun 2003;300:674. 12507502 10.1016/s0006-291x(02)02864-4

[14] Fujinami A Obayashi H Ohta K Enzyme-linked immunosorbent assay for circulating human resistin: resistin concentrations in normal subjects and patients with type 2 diabetes. Clin Chim Acta 2004;339:57–63. 14687894 10.1016/j.cccn.2003.09.009

[15] Silha JV Krsek M Skrha JV Plasma resistin, adiponectin and leptin levels in lean and obese subjects: correlations with insulin resistance. Eur J Endocrinol 2003;149:331–5. 14514348 10.1530/eje.0.1490331

[16] Zaidi SI Shirwany TA Relationship of serum resistin with insulin resistance and obesity. J Ayub Med Coll Abbottabad 2015;27:552–5. 26721005

[17] Sokhanguei Y Eizadi M Goodarzi MT Association of adipokine resistin with homeostasis model assessment of insulin resistance in type II diabetes. Avicenna J Med Biochem 2015;3: e26467.

[18] Menzaghi C Bacci S Salvemini L Serum resistin, cardiovascular disease and all-cause mortality in patients with type 2 diabetes. PLoS One 2013;8: e64729. 10.1371/journal.pone.0064729 PMC3670852 23755138

[19] American Diabetes Association. Type 2 diabetes in children and adolescents. Pediatrics 2000;105:671–80. 10699131 10.1542/peds.105.3.671

[20] Grarup N Sandholt C Hansen T Genetic susceptibility to type 2 diabetes and obesity: from genome-wide association studies to rare variants and beyond. Diabetologia 2014;57:1528. 24859358 10.1007/s00125-014-3270-4

[21] DIAbetes Genetics Replication and Meta-analysis (DIAGRAM) Consortium. Genome-wide trans-ancestry meta-analysis provides insight into the genetic architecture of type 2 diabetes susceptibility. Nat Genet 2014;46:234. 24509480 10.1038/ng.2897 PMC3969612

[22] Bonora E Targher G Alberiche M Homeostasis model assessment closely mirrors the glucose clamp technique in the assessment of insulin sensitivity: studies in subjects with various degrees of glucose tolerance and insulin sensitivity. Diabetes Care 2000;23:57. 10857969 10.2337/diacare.23.1.57

[23] Albareda M Rodríguez-Espinosa J Murugo M Assessment of insulin sensitivity and beta-cell function from measurements in the fasting state and during an oral glucose tolerance test. Diabetologia 2000;43:1507. 11151759 10.1007/s001250051561

[24] Reaven GM HOMA-beta in the UKPDS and ADOPT. Is the Natural History of Type 2009;2133–8. 10.1177/1479164109336038 20368203

[25] Friedewald W Levy R Fredrickson D Estimation of the concentration of low-density lipoprotein cholesterol in plasma, without use of the preparative ultracentrifuge. Clin Chem 1972;18:499. 4337382

[26] McTernan PG Fisher FM Valsamakis G Resistin and type 2 diabetes: regulation of resistin expression by insulin and rosiglitazone and the effects of recombinant resistin on lipid and glucose metabolism in human differentiated adipocytes. J Clin Endocrinol Metab 2003;88:6098–106. 14671216 10.1210/jc.2003-030898

[27] Fujinami A Obayashi H Ohta K Enzyme-linked immunosorbent assay for circulating human resistin: resistin concentrations in normal subjects and patients with type 2 diabetes. Clin Chim Acta 2004;339:57. 14687894 10.1016/j.cccn.2003.09.009

[28] Silha J Krsek M Skrha J Plasma resistin, adiponectin and leptin levels in lean and obese subjects: correlations with insulin resistance. Eur J Endocrinol 2003;149:331. 14514348 10.1530/eje.0.1490331

[29] Youn B Yu K Park H Plasma resistin concentrations measured by enzyme-linked immunosorbent assay using a newly developed monoclonal antibody are elevated in individuals with type 2 diabetes mellitus. J Clin Endocrinol Metab 2004;89:150. 14715842 10.1210/jc.2003-031121

[30] Costandi J Melone M Zhao A Human resistin stimulates hepatic overproduction of atherogenic ApoB-containing lipoprotein particles by enhancing ApoB stability and impairing intracellular insulin signaling. Circ Res 2011;108:727. 21293001 10.1161/CIRCRESAHA.110.238949

[31] Osawa H Onuma H Ochi M Resistin SNP-420 determines its monocyte mRNA and serum levels inducing type 2 diabetes. Biochem Biophys Res Commun 2005;335:596. 16087164 10.1016/j.bbrc.2005.07.122

[32] Osawa H Tabara Y Kawamoto R Plasma resistin, associated with single nucleotide polymorphism-420, is correlated with insulin resistance, lower HDL cholesterol, and high-sensitivity C-reactive protein in the Japanese general population. Diabetes Care 2007;30:1501–6. 17384338 10.2337/dc06-1936

[33] El-Shal A Pasha H Rashad N Association of resistin gene polymorphisms with insulin resistance in Egyptian obese patients. Gene 2013;515:233. 23178185 10.1016/j.gene.2012.09.136

